# Acute Renal Dysfunction and Candidemia due to Bilateral Ureteral Obstruction by *Candida albicans* Fungus Balls—Case Report

**DOI:** 10.1002/ccr3.72027

**Published:** 2026-02-13

**Authors:** Mizuki Kasahara, Fumito Yamabe, Masato Uetani, Hideyuki Kobayashi, Koichi Nakajima, Yozo Mitsui

**Affiliations:** ^1^ Department of Urology Toho University Faculty of Medicine Tokyo Japan

**Keywords:** acute kidney injury, fungi, hydronephrosis, urinary tract infection

## Abstract

Presented here is an extremely rare case of bilateral ureteral obstruction due to *Candida albicans* fungus balls, which led to acute kidney dysfunction and candidemia. An 83‐year‐old man was brought to our hospital after falling due to poor physical condition. He had been receiving abiraterone acetate for 1 month for metastatic castration‐resistant prostate cancer, while past medical history included type II diabetes, cardiovascular disease, and dementia. Blood test results revealed severe liver dysfunction, though whole‐body computed tomography (CT) findings showed no abnormalities. Based on the recent therapy course, the patient was diagnosed with drug‐induced liver damage caused by abiraterone acetate, and steroid pulse therapy and antibiotic administration were started. On Day 11 after starting that treatment, decreased urine output and renal dysfunction were noted. CT scanning revealed bilateral hydronephrosis and slightly dense masses at the origin of ureteral obstruction on both sides. Subsequently, *C. albicans* was detected in blood and urine samples, thus fungus balls were determined as the cause of bilateral hydronephrosis. Temporary hemodialysis was required, though clinical symptoms and biochemical findings gradually improved following insertion of bilateral ureteral stents and administration of antifungal therapy, and the patient was discharged 72 days after admission.

## Key Clinical Message

Pyelonephritis and fungemia associated with fungus balls are often asymptomatic and difficult to diagnose. Delayed or incorrect treatment results in life threatening. We Clinicians must keep in mind the possibility of a fungal infection especially in the patients with DM, immunosuppression, chronic disease, or malignancy.

## 1 Introduction

Funguria, also known as candiduria, often occurs in hospitalized patients. Generally, most cases are not caused by a true urinary tract infection, thus are asymptomatic and do not require treatment [1]. However, the presence of a symptomatic fungal urinary tract infection (UTI) will cause deterioration of the general condition. Candiduria has the potential to progress into life‐threatening candidemia, such as in patients with urinary system obstruction [2] and it has been reported that the in‐hospital mortality rate of such patients is significantly higher than that of those without candidemia [3]. Multidisciplinary treatment including prompt administration of antifungal drugs is thus required.

Although rare, fungal UTI can result in fungus ball formation leading to the obstruction of the urinary tract. Subsequent development of urinary retention and hydronephrosis is possible, which may lead to such conditions as pyelonephritis, fungemia, or kidney damage [4, 5, 6]. Reported here is an extremely rare case of a castration‐resistant prostate cancer (CRPC) patient who developed acute renal dysfunction and candidemia due to obstruction of the bilateral ureters by *Candida albicans* fungus balls.

## 2 Case History/Examination

An 83‐year‐old male CRPC patient who regularly visited the urology department of our hospital was rushed to the emergency room after falling because of poor physical condition and a head bruise. His medical history included ischemic heart disease, mild dementia, and well‐controlled type II diabetes mellitus (DM), with abiraterone acetate and steroid combination therapy started approximately 1 month earlier for CRPC with multiple lung metastases. Physical findings revealed a temperature of 40° and head abrasions, while a computed tomography (CT) examination of the whole body including head showed no abnormal results. Blood testing indicated mild pancytopenia, with a white blood cell count of 2700, hemoglobin of 10.6 g/dL, and platelet count of 103,000, along with an elevated inflammatory response shown by C‐reactive protein (CRP) at 5.07 mg/dL. Additionally, severe liver dysfunction was shown by aspartate aminotransferase (AST) and alanine aminotransferase (ALT) levels of 1243 and 540 IU/L, respectively. Although prothrombin time was prolonged (prothrombin activity 54%, prothrombin time/international normalized ratio 1.4), renal function remained normal with a serum creatinine level of 0.91 mg/dL. A few days prior to falling, a blood test performed the second week after administering abiraterone showed no abnormal results. Based on patient condition, blood test results, and treatment progress, the diagnosis was drug‐induced liver injury caused by abiraterone acetate and he was urgently admitted for treatment.

**FIGURE 1:**
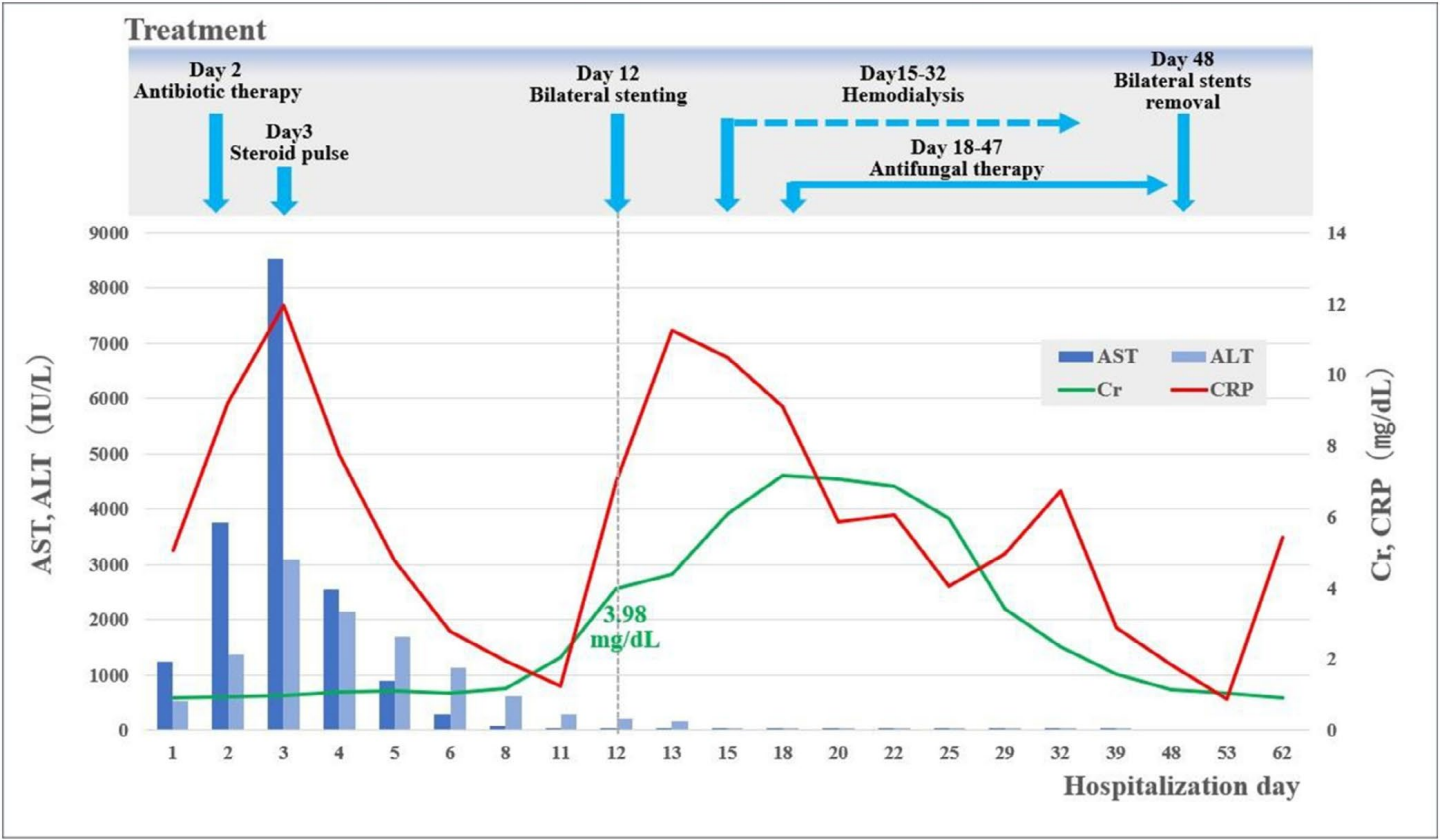
Clinical course following admission.

**FIGURE 2:**
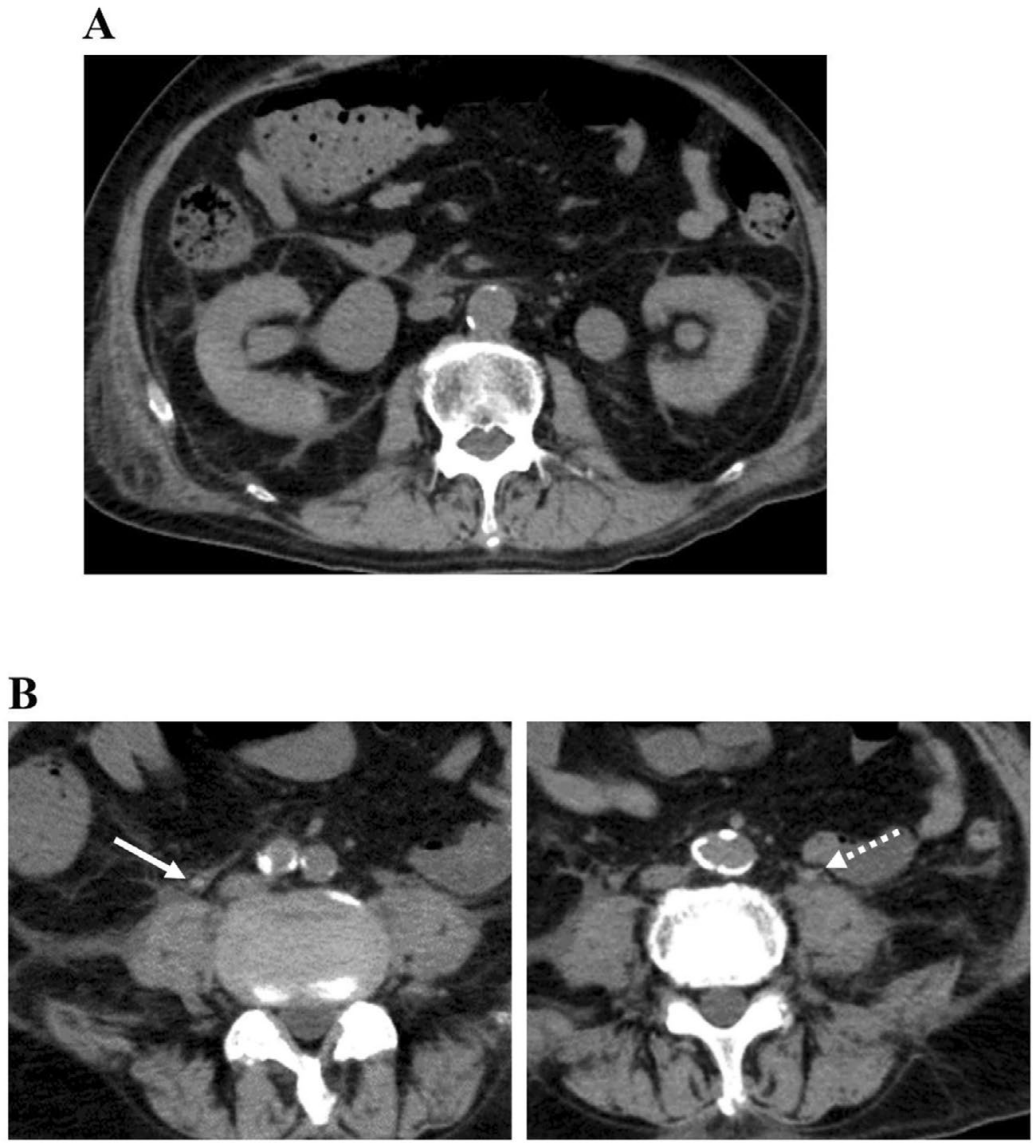
Abdominal non‐contrast CT scan images obtained for examination of worsened renal function. The causes of obstruction were (A) bilateral hydronephrosis and (B) presence of slightly dense masses in the examined sites (solid line arrow indicates right side, dotted arrow indicates left side).

Central venous (CV) and urinary catheters were inserted, and the patient was managed in the advanced care unit. The clinical course following admission is shown in Figure 1. Intravenous fluids and antibiotic treatment with meropenem were immediately administered. However, worsening liver dysfunction was observed, thus steroid pulse therapy was started on the third day after admission. Serum AST and ALT levels increased to a maximum of > 8000 and > 3000 IU/L, respectively. Following the start of treatment, liver enzyme and CRP levels tended to quickly decrease, thus steroid pulse therapy could be terminated within 7 days. On day 11 of hospitalization, laboratory tests showed serum creatinine increased to 2.06 mg/dL, which worsened to 3.98 mg/dL the next day, along with decreased urine output and increased CRP (7.0 mg/dL). Subsequent abdominal non‐contrast CT scanning confirmed bilateral hydronephrosis and showed slightly dense masses at the site of obstruction on both sides (Figure 2).

## 3 Differential Diagnosis, Investigations, and Treatment

Postrenal renal failure due to urinary lithiasis was suspected, thus a bilateral double J ureteral stent was immediately inserted. It was possible to place the stent on both sides without resistance, with cloudy urine and a small amount of soft tissue mass observed flowing from the stent. Bilateral hydronephrosis was resolved, though there was no improvement in renal function, suggesting that factors other than postrenal were involved. On hospitalization Day 15, the patient had become nearly anuric and serum creatinine reached 6.10 mg/dL, thus hemodialysis was started. Blood and urine cultures confirmed *C. albicans* growth and intravenous antifungal therapy with fosfluconazole (100 mg on Days 1 and 2, 50 mg thereafter) was started 18 days after admission. The CV catheter was immediately removed and a tip culture performed, with negative results. In addition, chest CT and fundus examinations showed no findings suggestive of fungal infection, while nasal bacterial culture findings were also negative. Based on the clinical course and these findings, it was concluded that a retrograde infection from urine caused candidemia, while the slightly dense masses in both ureters shown by CT were speculated to be fungus balls.

**FIGURE 3:**
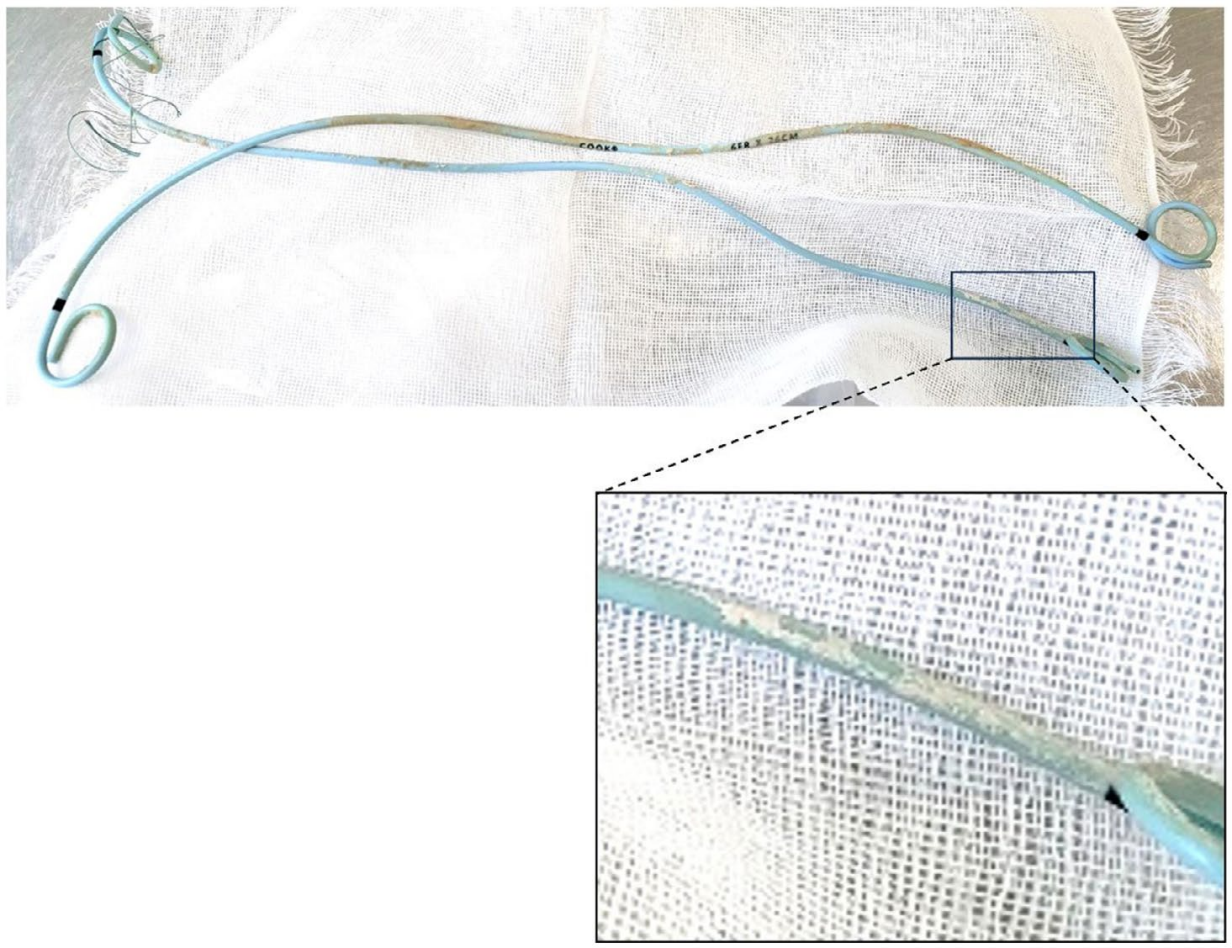
Bilateral ureteral double‐J stent following removal. A portion of the ureteral stent removed after treatment was covered with a yellowish‐white sticky substance. Subsequent catheter cultures identified *Candida albicans* .

With every other day hemodialysis and antifungal treatment, serum creatinine and inflammatory parameters gradually decreased, and patient condition showed remarkable improvement. Hemodialysis was completed after 17 sessions and the antifungal drug was switched to oral administration, then stopped on treatment Day 29. During the treatment period, repeated blood cultures revealed disappearance of *C. albicans* , though the fungus remained in urine cultures. Two months after starting treatment, the bilateral ureteral catheters were removed and found to be covered with a yellowish‐white sticky substance, with *C. albicans* identified in catheter culture results (Figure 3). Abdominal CT scanning performed after removal of the bilateral ureteral catheters showed no recurrence of bilateral hydronephrosis, and the patient was discharged after 72 days of hospitalization with good treatment progress.

## 4 Discussion

*Candida* fungus balls, formations of fungal hyphae, are typically associated with DM, immunosuppression, chronic disease, or malignancy, except for rare reports of occurrence in healthy individuals [4, 5, 6, 7]. Once formed, they can cause candidemia due to obstruction of the urinary tract, leading to progression of a fulminant disease with high lethality [4]. Furthermore, it should be noted that patients with only one kidney are likely to subsequently experience acute renal dysfunction [5]. In the present case, it is speculated that in addition to DM history, the immune system was weakened by high‐dose steroid administration for drug‐induced liver damage, thus promoting candidemia and fungus ball formation within a short period of time. The possibility of hematogenous spread of candidemia was ruled out based on the CV catheter and sputum culture results, as well as chest CT findings, and it was considered that obstruction of the upper urinary tract by the fungus balls eventually caused candidemia via a urological retrograde infection route. An interesting aspect of this case is their simultaneous bilateral occurrence. Acute renal dysfunction in both kidneys was due to a combination of postrenal factors caused by the fungus balls, as well as others such as infection and general condition deterioration. Only three known cases of renal failure induced by fungal balls in bilateral ureters have been reported including the present while this is the first with subsequent candidemia [8, 9].

Specific imaging results indicating urinary tract fungal balls have not been established as there are very few reported cases, making early diagnosis challenging. Indeed, most previous reports have only noted them as an atypical filling defect showing radiolucency in excretory or retrograde urography findings. In the present case, slightly dense masses in the bilateral ureters were identified by non‐contrast CT, then later diagnosed as fungus balls based on urine culture results. Similarly, Lee reported fungal balls observed in CT images as stone‐like lesions with areas of high attenuation and pointed out that such high‐density findings may be due to deposits on the fungal balls, such as bacterial calcification [10]. CT findings of fungus balls that mimicked those of urinary stones have also been reported [11]. Notably, Arichi et al. indicated that T2‐weighted magnetic resonance imaging can be useful for diagnosis [5]. A comprehensive understanding of the clinical course associated with fungal balls along with possible imaging findings is important for prompt diagnosis.

Fungus balls are generally treated with systemic antifungal drugs, with minimally invasive or surgical removal also sometimes performed. Effectiveness of irrigation of the urinary duct system with amphotericin B has been demonstrated in previous studies [4, 5, 8]. Furthermore, surgery using nephroscopy can be effective for large percutaneous fungus ball removal [12]. Based on clinical data, the infectious Disease Society of America Clinical Practice Guideline for the Management of Candida suggests that management of patients with *Candida* UTIs associated with fungus balls can include surgery, systemic antifungals, and amphotericin B irrigation via a nephrostomy tube [13]. Fortunately, those in the present case disappeared, while the resultant urinary tract obstruction was relieved only by systemic administration of antifungal drugs, which may have been due to the small size of the fungal balls or ureteral stent insertion.

Most fungal infections, both symptomatic and asymptomatic, can be considered as iatrogenic disease. Drug‐induced liver injury caused by abiraterone acetate, requiring steroid pulse therapy, was thought to be the initial cause of the fungal infection in the present case. Although extremely rare, based on reports of fulminant liver failure due to abiraterone acetate, caution is required in medical practice [14].

## 5 Conclusions

Fungus balls can be easily misdiagnosed as urolithiasis, resulting in delayed or incorrect treatment. Most urinary fungal infections are asymptomatic, though when associated with pyelonephritis and fungemia, they can cause morbidity or mortality. Clinicians must keep in mind the possibility of a fungal infection based on the treatment course and seek diagnosis and perform appropriate treatment without delay.

## Author Contributions

**Mizuki Kasahara:** conceptualization, investigation, visualization, writing – original draft, writing – review and editing. **Fumito Yamabe:** writing – review and editing. **Masato Uetani:** writing – review and editing. **Hideyuki Kobayashi:** writing – review and editing. **Koichi Nakajima:** writing – review and editing. **Yozo Mitsui:** resources, supervision, writing – review and editing.

## Funding

The authors have nothing to report.

## Consent

Written informed consent was obtained from the patient to publish this case report in accordance with the journal's patient consent policy.

## Conflicts of Interest

The authors declare no conflicts of interest.

## Data Availability

Data available on request from the authors.
